# Quantum transport in a chain of quantum dots with inhomogeneous size distribution and manifestation of 1D Anderson localization

**DOI:** 10.1038/s41598-020-73578-z

**Published:** 2020-10-07

**Authors:** Moon-Hyun Cha, Jeongwoon Hwang

**Affiliations:** 1 School of Engineering, Brown University, Providence, RI 02912 USA; 2 CSE Team, Data and Information Technology Center, Samsung Electronics, Hwaseong, 18448 Republic of Korea; 3 Department of Physics Education, Chonnam National University, Gwangju, 61186 Republic of Korea

**Keywords:** Electronic properties and materials, Quantum dots, Applied physics

## Abstract

The effect of inhomogeneous quantum dot (QD) size distribution on the electronic transport of one-dimensional (1D) QD chains (QDCs) is theoretically investigated. The non-equilibrium Green function method is employed to compute the electron transmission probabilities of QDCs. The ensemble averaged transmission probability shows a close agreement with the conductivity equation predicted by Anderson et al. for a disordered electronic system. The fidelity of quantum transport is defined as the transmission performance of an ensemble of QDCs of length *N* (*N*-QDCs) to assess the robustness of QDCs as a practical electronic device. We found that the fidelity of inhomogeneous *N*-QDCs with the standard deviation of energy level distribution *σ*_*ε*_ is a Lorentzian function of variable *Nσ*_*ε*_^2^. With these analytical expressions, we can predict the conductance and fidelity of any QDC characterized by (*N*, *σ*_*ε*_). Our results can provide a guideline for combining the chain length and QD size distributions for high-mobility electron transport in 1D QDCs.

## Introduction

Electronic quantum transport through an array of quantum dots (QDs) has been an interesting research topic for low-dimensional systems at low temperature. Experimentally, a long-range ordered array of QDs, or QD solid, can be synthesized from colloidal QDs^1–7^. One interesting aspect of QD solid is that the coupling strength between QDs can be continuously tuned by adjusting the interdot separation and surface passivation^8,9^. This controllability allows the engineering of optical, electrical, thermal, and mechanical properties of QD solids^2^. With QD as a building block, bottom-up fabrication of electronic and optoelectronic devices with the desired properties may be possible.

Notably, even the best synthesis method for colloidal QDs can produce QDs with a 3%-5% standard deviation in size^2^. Molecular beam epitaxy-grown QD arrays also exhibited a Gaussian statistical distribution in dot size^10,11^. While previous theoretical studies on QDs mainly examined the electronic properties of small QD systems^12–17^, a recent study examined the effect of impurity QDs on the electron transport in two-dimensional (2D) QD solids, in which the impurity QD was introduced as a perturbation to a periodic potential^18^. However, a systematic analysis on the quantum transport of an array of QDs with inhomogeneous size distribution is still lacking.

When QDs are different only in size, the statistical distribution of the dot size is converted into a statistical distribution of quantized QD energy levels, originating from the quantum-size effect by the relation $E \propto \frac{1}{{r^{2} }}$ in the case of a spherical QD^14^. In QD solids, the relative size of the QD affects the relative position of the energy level, such as the lowest unoccupied molecular orbital (LUMO) 1s level, between adjacent QDs. As can be seen from the layered 2D heterostructures, the electrical contact type and electrical performance are greatly affected by the band alignment, i.e., difference in band gap and band edge positions. Similarly, the electronic properties of QD solids will be influenced by the relative size of the constituent QDs.

QDs can be integrated into a desired configuration^3,5^, and QD solids with high electron mobility can be applied to the channel material of field effect transistors. In addition to the low cost and solution processability over a large area^1–3^, the compatibility with flexible and stretchable substrates makes QD solids beneficial for device application ^3^. If it is unavoidable to have a size distribution in QDs during a synthesis, it is important to know how much inevitable randomness quantitatively changes the transport properties for electrical performance control.

In this study, we investigate quantum transport in one-dimensional (1D) chains of non-uniform QDs by calculating the transmission probability. The non-equilibrium Green function (NEGF) method is employed for calculating the transmission probability spectra. First, a conductance measure is defined and calculated to evaluate the transport capability of individual QD chains (QDCs). Subsequently, we explore the effect of the chain length and energy level distribution on the transport performance of an ensemble of QDCs. It is found that the ensemble-averaged transmission probability exhibits the same analytical behavior predicted for disordered electronic systems^19^. Finally, for an ensemble of QDCs, we calculate the proportion of ensemble members with the conductance measure higher than a criterion, which is defined as the fidelity of the QDC and found to be a Lorentzian function of the chain length and variance of the energy level distribution. Thus, the system size (length) and size variance of constituent QDs carry the information about ensemble-averaged conductance and fidelity. Our theoretical study can provide an in-depth understanding of QDCs with inhomogeneous QD size distributions and suggest ways to manipulate their electrical properties.

## Results and discussion

We compute transmission probability through QDCs as a function of energy in a coherent tunneling regime. The Hamiltonian of an *N*-QDC and contacting (left and right) leads is given by $H = H_{QDC} + H_{L} + H_{R} + H_{T} .$ Here, $H_{QDC}$ is the Hamiltonian of the QDC:

1 $$ H_{QDC} = \mathop \sum \limits_{i = 1}^{N} \varepsilon_{i} d_{i}^{\dag } d_{i} + \mathop \sum \limits_{i = 1}^{N - 1} t_{i} \left( {d_{i + 1}^{\dag } d_{i} + H.c.} \right), $$

where $d_{i}^{\dag }$ creates an electron in the *i*th QD and $t_{i}$ is the interdot tunneling matrix element. We neglect the interaction between electrons to solely focus on the effect of the randomness of energy levels. $H_{L} \left( {H_{R} } \right)$ describes the left (right) electrode as a non-interacting electron gas system:

2 $$ H_{\alpha } = \sum \limits_{k} \varepsilon_{k\alpha } a_{k\alpha }^{\dag } a_{k\alpha } ,{{ \upalpha }} = L\,\, {\text{or }}\,\,R, $$

where $a_{kL}^{\dag } \left( {a_{kR}^{\dag } } \right)$ creates an electron of momentum *k* in the left (right) electrode. Finally, the tunneling Hamiltonian between the QDC and electrodes is given as

3 $$ H_{T} = \mathop \sum \limits_{k} t_{k1} d_{1}^{\dag } a_{kL} + \mathop \sum \limits_{k} t_{kN} d_{N}^{\dag } a_{kR} + H.c., $$

where the tunneling matrix element $t_{k1} \left( {t_{kN} } \right)$ describes the coupling between the left (right) electrode and the first (last) QD. The transmission probability of an electron of energy ε is given as ^20^

4 $$ T\left(\epsilon \right) = Tr\left( {{\Gamma }_{L} G_{S}^{a} \left(\epsilon \right){\Gamma }_{R} G_{S}^{r} \left(\epsilon \right)} \right), $$

where $G_{S}^{r} = \left[ {\left(\epsilon { + i\eta } \right)I - H_{QDC} - {\Sigma }_{L} - {\Sigma }_{R} } \right]^{ - 1}$ is the retarded one-particle Green’s function of the system of QDs and the advanced Green’s function corresponds to the Hermitian conjugate of the retarded one, i.e. $G_{S}^{a} = \left( {G_{S}^{r} } \right)^{\dag }$. The broadening function ${\Gamma }_{L\left( R \right)} = i\left( {{\Sigma }_{L\left( R \right)} - {\Sigma }_{L\left( R \right)}^{\dag } } \right) = - 2Im\left( {{\Sigma }_{L\left( R \right)} } \right),$ where $\Sigma_{L\left( R \right)}$ is the self-energy of the QDC arising from its coupling to the left (right) electrode and ${\Gamma } = {\Gamma }_{L} = {\Gamma }_{R}$ in this study. A zero-voltage quantum conductance is directly proportional to the transmission probability based on the Landauer formula ^21^, i.e., the conductance $G = \frac{{e^{2} }}{h}T$ for a single eigen channel.

**Figure 1:**
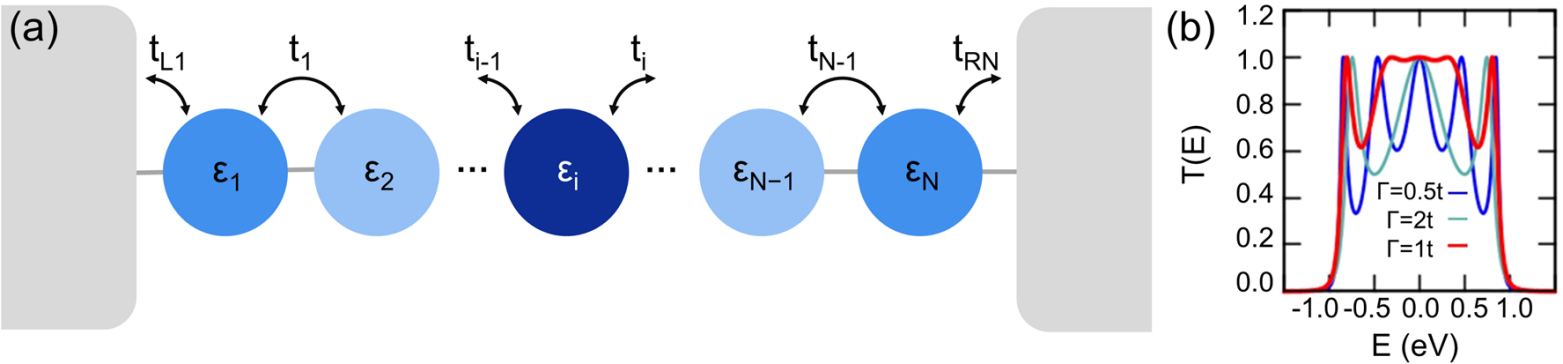
(**a**) Schematic description of a 1D chain of *N* QDs in contact with two metallic leads. Different colors represent different energies (i.e., sizes). (**b**) Dependence of dot-lead coupling Γ on the transmission probability of a uniform (i.e., ${\varepsilon }_{i}={\varepsilon }_{0})$ 5-QDC with the tunneling matrix element *t* = 0.5 eV.

Figure 1(a) illustrates a QDC with varying energies, where the different energy levels of QDs are presented as circles with different colors. First, we consider a chain of uniform *N* QDs (uniform *N*-QDC), where *N* is the number of serially connected QDs in the chain. By uniform QDC, we mean $\varepsilon_{i} = \varepsilon_{0} = 0$ for all dots and $t_{i} = t_{0}$ for all pairs of nearest neighbors. For *N* = 5, our calculation results agree well with the previous studies^22,23^ as shown in Fig. 1(b). When the energy distribution is introduced, Γ = 1*t* gives the maximum transmission probability; therefore, Γ = 1*t* is selected for this study. The magnitude of $t_{0}$ only affects the band width in a uniform manner, that is, the width of the calculated transmission functions is uniformly scaled in energy (see Supplementary Information for details).

**Figure 2:**
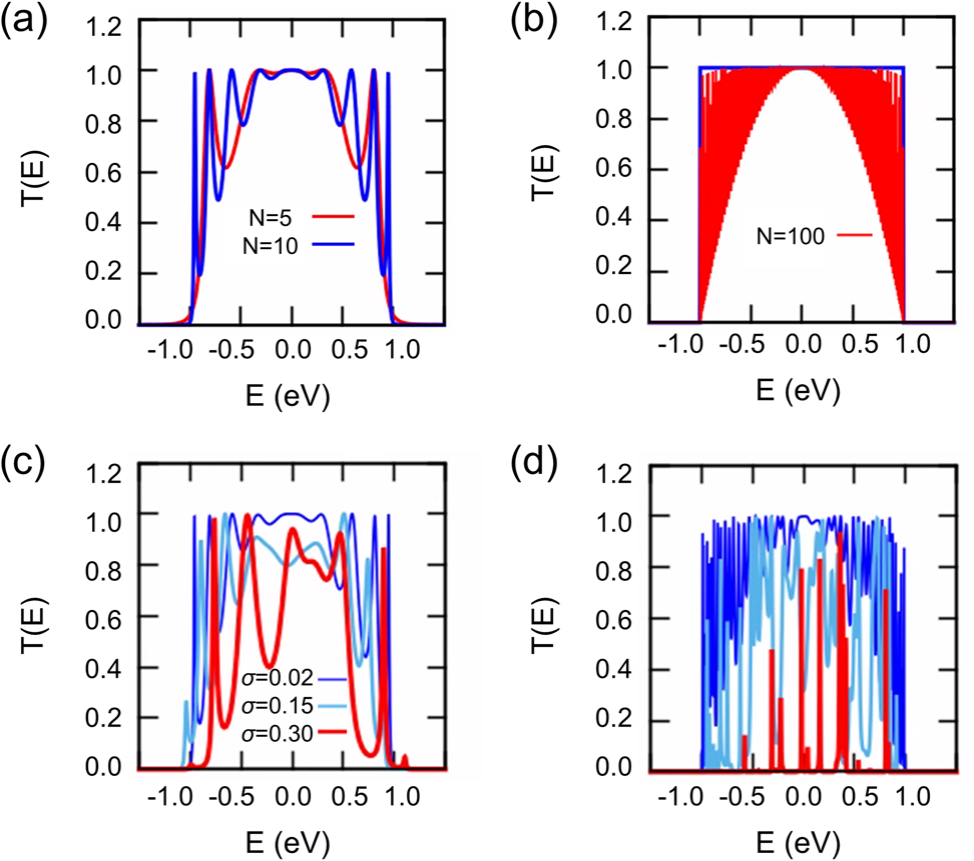
Effect of system size and energy variance on transmission probability. (**a**) Transmission probability of uniform QDC for *N* = 5 and 10. (**b**) As *N* increases, a line connecting the maxima of transmission probability peaks approaches that of an infinite QDC, shown as the blue line. Transmission probability of typical QDCs with *σ*_*ε*_ = 0.02, 0.15, and 0.3 for (**c**) *N* = 10 and (**d**) *N* = 50, respectively^26^.

Then, we consider QDCs with a dot-size distribution. QDCs can be specified with two parameters, i.e., *N* and *σ*_*ε*_, the standard deviation of the energy level distribution. To examine the chain length effect, we first compute the transmission probability of uniform *N*-QDCs for *N* between 5 and 100, and the results are shown in Fig. 2(a) and (b). As *N* increases, the number of peaks symmetrically increases with respect to zero energy, and a line connecting the maxima of peaks approaches that of an infinite QDC without a contact effect (blue line). With uniform QDs, increasing *N* does not degrade the transmission probability. Next, a Gaussian-type energy distribution is introduced with a mean value of 0 eV. We use the same value of ${t}_{i}={t}_{0}$ for all pairs as a first approximation. For spherical QDs, we may assume hydrogenic electronic states, the energy level (E) of which is related to its radius (r) by $E\propto \frac{1}{{r}^{2}}$^14^. For a small size variation ($\Delta r<r$), the energy deviation can be expressed as ${E}^{^{\prime}}\propto \frac{1}{{\left(r+\Delta r\right)}^{2}}\sim \frac{1}{{r}^{2}}\left(1-\frac{2\Delta r}{r}\right)$, i.e., ${E}^{^{\prime}}\sim E\left(1-\frac{2\Delta r}{r}\right)$. The reported size deviation of 5% can be converted into an energy deviation of 10%. For instance, for a PbS QD system, the LUMO level binding energy of which is approximately 4 eV^24^, the 10% standard deviation in energy is *σ*_*ε*_ = 0.4 (eV). However, for QDs of different sizes, *σ*_*ε*_ of 0.4 eV can be either greater or smaller than a 10% (5%) standard deviation in energy (size).

A QDC of (*N*, *σ*_*ε*_) corresponds to randomly collected (and randomly arranged) *N* QDs among a bunch of QDs of which the statistical distribution is meaningful. Considering the fact that the coupling strength between QDs can be continuously tuned ^2^, the tunneling matrix element *t* is fixed as 0.5 eV in the remaining part of the study. For each *N* (i.e., 5, 10, 20, 30, 40, and 50), an ensemble of QDCs (more than 4000 samples when *N* = 5–20 and more than 2000 samples when *N* = 30–50) is generated with varying *σ*_*ε*_ (from 0.02 to 0.5). The computed transmission probability functions of the arbitrarily chosen QDCs of *N* = 10 (*N* = 50) with *σ*_*ε*_ = 0.02, 0.15, and 0.3 are shown in Figs. 2(c) and (d), respectively. As *σ*_*ε*_ increases, the area under the transmission probability function is reduced, indicating a lowered transport capability. For an ensemble calculation, a set of pseudo-random numbers with a Gaussian distribution is generated via Python Standard Library ^25^ (See Supplementary Information Fig. S2 for more discussion).

**Figure 3:**
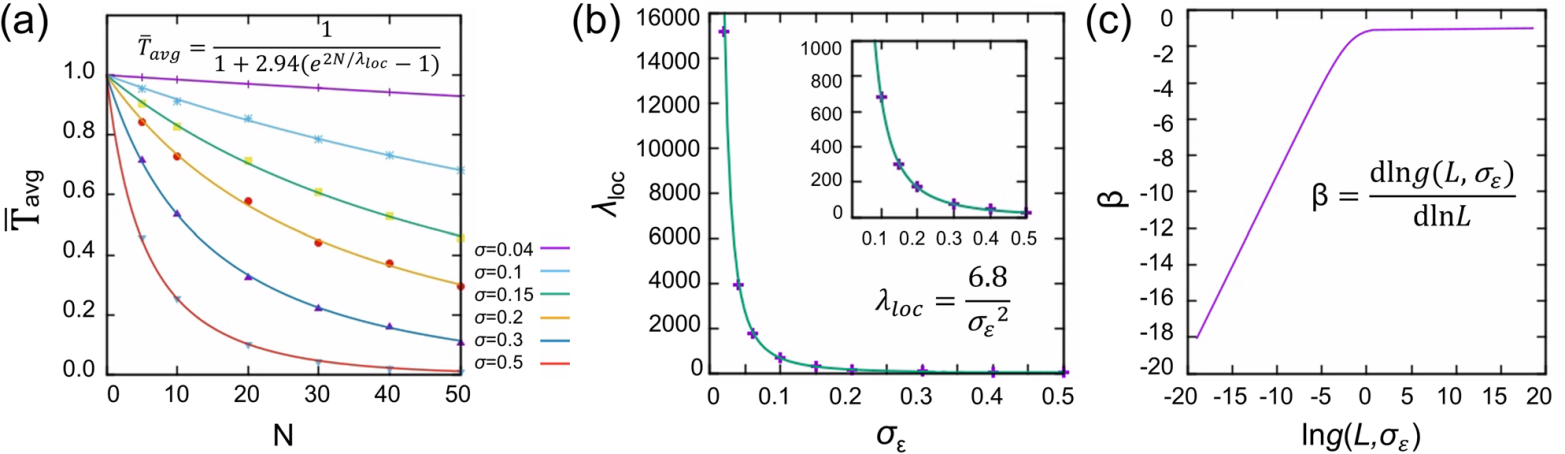
Manifestation of 1D Anderson localization in quantum dot chains with inhomogeneous size distribution. (**a**) Ensemble-averaged $\bar{T}$ (marked as points) for each *σ*_*ε*_ follows the same analytical behavior $\frac{1}{1+\alpha ({e}^{2N/{\lambda }_{loc}}-1)}$ (shown as solid lines) predicted by Anderson et al*.*^19^ with a different resistivity constant (i.e., α = 2.94). (**b**) The localization length is inversely proportional to *σ*_*ε*_^2^, which is plotted in the unit of QD size. The inset is the magnified version for a clear view. (**c**) Asymptotic forms of the scaling function β(g) agree well with the prediction of Abrahams et al*.*^27^ for dimension *d* < 2, where *g* is the conductivity and *L* is the system size (i.e., *N*, in our case)^26^.

To evaluate the transport capability of individual QDCs, we define a conductance measure. While the transmission function is calculated at zero temperature, we may include thermal effect in the transmission probability around the Fermi level as $\bar{\text{T}}\equiv \frac{\int d\epsilon \mathrm{ f}\left(\epsilon \right)\left(1-\mathrm{f}\left(\epsilon \right)\right)\cdot \mathrm{T}\left(\epsilon \right)}{\int d\epsilon \mathrm{f}\left(\epsilon \right)\left(1-\mathrm{f}\left(\epsilon \right)\right)}$, where $\mathrm{f}\left(\epsilon \right)$ is the Fermi–Dirac distribution. By approximating f(1 − f) as a rectangular function with a width of 2kT centered at ${\epsilon }_{\mathrm{F}}$, which is assumed to be 0 eV in this study, $\bar{\text{T}}$ can be approximated to $\frac{{\int }_{-\mathrm{kT}}^{\mathrm{kT}}d\epsilon \mathrm{ T}\left(\epsilon \right)}{{\int }_{-\mathrm{kT}}^{\mathrm{kT}}d\epsilon }$. If we take kT to be the thermal energy scale at room temperature, $\bar{\text{T}}=\frac{{\int }_{-0.025 eV}^{0.025 eV}T\left(\epsilon \right)d\epsilon }{0.05 eV}$. For any choice of kT, the maximum value of $\bar{\text{T}}$ is 1 for a transparent transport. The ensemble-averaged $\bar{\text{T}}$ is shown in Fig. 3(a) as a function of *N*, which clearly shows that that the conductance measure decreases faster with the chain length when ${\upsigma }_{\upvarepsilon }$ is larger. Notably, our numerical results exhibit the same analytical behavior predicted by Anderson et al., where resistivity is predicted by scaling theory^19^. Specifically, we obtain

5 $$ \bar{T}\left( {N,\sigma_{\varepsilon } } \right) = \frac{1}{{1 + \rho \left( {N,\sigma_{\varepsilon } } \right)}}, $$

where resistivity $\rho \left(N,{\sigma }_{\varepsilon }\right)=\mathrm{\alpha }({e}^{2N/{\lambda }_{loc}}-1)$ with resistivity constant α and localization length *λ*_*loc*_, which are fitting parameters of our study. It is found that α is the same for all ${\sigma }_{\varepsilon }$, that is α = 2.94 as shown in Fig. 3(a), and *λ*_*loc*_ depends on ${\sigma }_{\varepsilon }$. From the analytical expression of $\bar{T}\left(N,\sigma \right),$ the localization length is related to the standard deviation of energy level distribution *σ*_*ε*_ by the relation ${\lambda }_{loc}=\frac{6.8}{{{\sigma }_{\varepsilon }}^{2}}$ (in the unit of QD size) as shown in Fig. 3(b). It can be interpreted that when the variance (${{\sigma }_{\varepsilon }}^{2})$ of energy level distribution is 6.8, the electronic state is localized on one QD. This relation clearly shows that the localization length decreases as the randomness of the system increases. Furthermore, the scaling theory of localization^27^ is applied to plot the scaling function β(g)$\equiv \frac{\mathrm{dln}g(L)}{\mathrm{dln}L}$, as shown in Fig. 3(c). In the scaling function β(g), g(L) is the conductivity (1/$\rho$) as a function of system size L (i.e., *N* in our case). Remarkably, β(g) follows the same asymptotic form predicted by Abrahams et al.^27^ for dimension *d* < 2; β(g) is linearly dependent on ln[g(L)] for small g ($\to 0)$ and asymptotically approaches a constant value *d* − 2 (= − 1 for one-dimensional system) for large g ($\to \infty )$.

**Figure 4:**
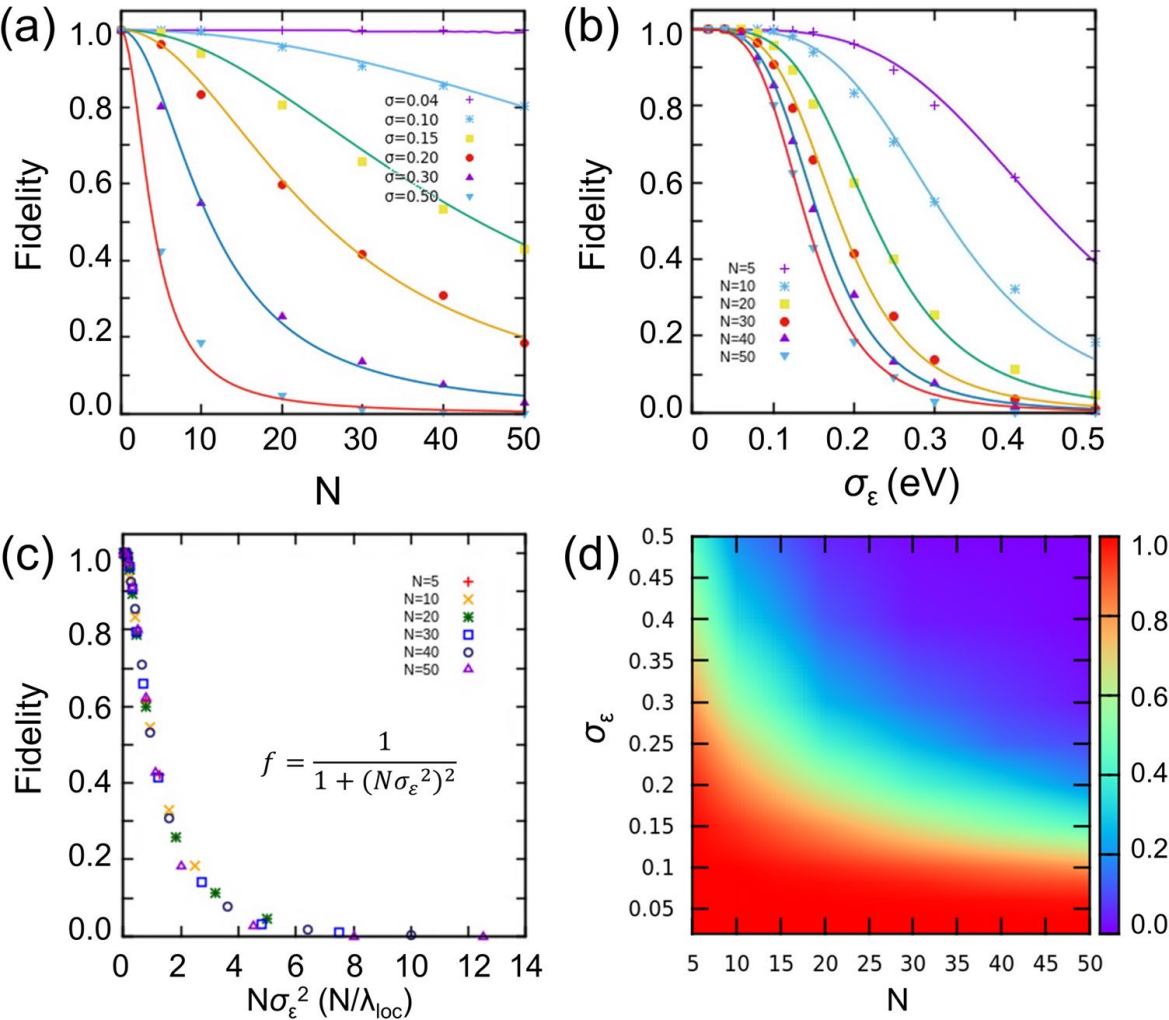
Fidelity of QDCs characterized by (*N*, *σ*_*ε*_). (**a**) Fidelity versus *σ*_*ε*_ and (**b**) fidelity versus *N*, which show that the longer the chain, the more susceptible it is to the size variations, and vice versa. The solid lines correspond to the analytical function $f\left(N,{\sigma }_{\varepsilon }\right)$ shown in (**c**). (**c**) Fidelity as a function of *Nσ*_*ε*_^*2*^ (~ *N/λ*_*loc*_). Calculated data of various *N*-QCDs for varying *σ*_*ε*_ fall into the same line. (d) 2D color map of fidelity with varying *N* (from 5 to 50) and *σ*_*ε*_ (from 0.02 to 0.5)^26^.

Now, we define the fidelity ($0\le f\le$ 1) of quantum transport in QDC as a proportion of members of the ensemble that have $\bar{T}$ higher than a criterion. This definition corresponds to a classification of systems into two groups exhibiting current on-state and off-state, where the criterion of 0.5 is chosen. In a statistical terminology, fidelity is a cumulative probability $P\left(\bar{T}>0.5\right)$ of a continuous variable $\bar{T}$. Analyses with the different choices of criterion value are presented in Fig. S3 and S4 of Supplementary Information. The fidelity of *N*-QDCs with *σ*_*ε*_ between 0 and 0.5 is calculated and presented in Fig. 4. Figure 4(a) and (b) clearly show that a longer chain is more susceptible to the size variations. Fidelity is higher than 0.8 for all *N* when ${\sigma }_{\varepsilon }\le 0.1$*,* but it shows a fast decrease with larger *σ*_*ε*_. This result indicates that for a highly transparent transport of relatively long QDC, it is necessary to synthesize QDs of nearly uniform energy levels with a standard deviation of 0.1 in energy or 1%–2% standard deviation in size. Interestingly, by fitting the calculated data points in Fig. 4(a) and (b), we obtain a simple analytical expression for fidelity as a function of *Nσ*_*ε*_^2^:

6 $$f\left(N,{\sigma }_{\varepsilon }\right)=\frac{1}{1+{(N{{\sigma }_{\varepsilon }}^{2})}^{2}},$$

which is a Lorentzian function of *Nσ*_*ε*_^2^ or *N/λ*_*loc*_ that can be interpreted as the system size (chain length) scaled by the localization length. If we plot fidelity as a function of *Nσ*_*ε*_^2^, all the data points of various *N*-QCDs for varying *σ*_*ε*_ fall into the same line, as shown in Fig. 4(c). To our surprise, this analytical expression makes it possible to predict the fidelity of any ensemble of QCDs characterized by (*N*, *σ*_*ε*_^2^), in addition to the ensemble-averaged $\bar{T}$. Interestingly, for different choices of the criterion value, the fidelity function retains the Lorentzian shape allowing us to predict fidelity of any ensemble of QCDs. A general expression for the relation between *N* and *σ*_*ε*_^2^ can be obtained from Eq. (6) as $N{{\sigma }_{\varepsilon }}^{2}=\frac{1-f}{f}$. For example, when the fidelity of *N*-QDCs is 0.5, the relation ${\sigma }_{\varepsilon }=\frac{1}{\sqrt{N}}$ holds. Moreover, at this point, $\bar{T}$ becomes 0.5 because *ρ* becomes 1 by Eq. (5). From the distribution of $\bar{T}$ (see Supplementary Information Fig. S5) ${\sigma }_{\varepsilon }=\frac{1}{\sqrt{N}}$ is estimated to be the transition point, where the majority of the ensemble population is reversed based on the median value $\bar{T}=0.5$. The relation between *N* and *σ*_*ε*_^2^ can also be recognized in the color map shown in Fig. 4(d), i.e., ${\sigma }_{\varepsilon }=\frac{1}{\sqrt{N}}\sqrt{\frac{1-f}{f}}$. We note that the relation ${\sigma }_{\varepsilon }=\frac{1}{\sqrt{N}}\sqrt{\frac{1-f}{f}}$ resembles the expression for the localization length ${\lambda }_{loc}=\frac{6.8}{{{\upsigma }_{\varepsilon }}^{2}}$, or ${\sigma }_{\varepsilon }=\frac{2.6}{\sqrt{{\lambda }_{loc}}}$.

We also examine the effect of sorting the order of QDs by size or energy. Assuming that the surface of QD is well passivated and the shapes of the QDs are the same, the energy level can be solely determined by the size of QD. For *N* = 5–30, we calculate the fidelity of QDCs that are ordered in the increasing QD size. In this case, the fidelity decreases much slowly with *σ*_*ε*_ as compared with the randomly arranged case. This result indicates that the energy level difference between coupled QDs (i.e., coupled by the tunneling matrix element) is an important parameter for modulating transport capability. Up to ${\sigma }_{\varepsilon }=0.3$ (~ 4% in size for the specific PbS QD), the fidelity is over 0.95, but it drastically decreases afterward (see Fig. S6). It can be understood that by sorting the energy levels in a monotonically increasing order, the energy difference between the leftmost (rightmost) QD and the left (right) leads is greatly enlarged while the energy level at the center of the chain is around the Fermi level. On the basis of this result, we can suggest a way to enhance the electrical performance of QDCs by a post physical process. That is, we may use a centrifuge to arrange QDC in an increasing size order (i.e., mass) and thus in a decreasing energy order.

Finally, the current analysis is rigorously applicable to serially aligned 1D QDCs. For the QD ribbons of finite width or 2D QD solids, the analytical expression for $\rho \left(N,{\sigma }_{\varepsilon }\right)$ will be the same up to a proportionality constant (i.e., $({e}^{2N/{\lambda }_{loc}}-1)$ with different *λ*_*loc*_) because it is the universal behavior of disordered electronic systems^19,27^; however, the resistivity constant $\mathrm{\alpha }$ may differ. At the same time, the requirements for high-mobility transport can be relieved in those systems because there can be several pathways to go through QD arrays. This is an issue for future studies to explore.

## Conclusion

We have investigated the effect of QD size distribution on quantum transport through 1D QDCs. Ensemble-averaged transmission probability exhibits 1D Anderson localization, which manifests itself in the analytic expression of averaged transmission probability and scaling behavior of conductivity. With the analytical expression, the ensemble-averaged conductance of *N*-QDC with any value of *σ*_*ε*_ can be predicted. Fidelity is defined to evaluate the transport performance of ensembles of QDCs. When *σ*_*ε*_ $(\le 0.1)$, all the systems show high fidelity even in a 1D structure where the transport path is quite limited. The fidelity of any *N*-QDC is a function of *Nσ*_*ε*_^2^ and can be predicted from a general expression. This study can provide an in-depth understanding of QDCs with inhomogeneous QD size distributions. Our finding is in good agreement with the predicted 1D localization properties, which indicates that 1D QDCs with an inhomogeneous size distribution can act as a material platform to realize the 1D localization of electron states. Moreover, our numerical results can provide a guideline for high-mobility electron transport in 1D QDCs, that is, *how uniform the QD sizes should be* for a given length of array, and suggest a way to construct artificial solids with varying electrical performances.

## Supplementary information

Supplementary file1

## References

[1] Kagan CR, Lifshitz E, Sargent EH, Talapin DV (2016). Building devices from colloidal quantum dots. Science.

[2] Kagan CR, Murray CB (2015). Charge transport in strongly coupled quantum dot solids. Nat. Nanotechnol..

[3] Yang J, Choi MK, Kim D-H, Hyeon T (2016). Designed assembly and integration of colloidal nanocrystals for device applications. Adv. Mater..

[4] Murray CB, Kagan CR, Bawendi MG (2000). Synthesis and characterization of monodisperse nanocrystals and close-packed nanocrystal assemblies. Annu. Rev. Mater. Sci..

[5] Whitham K (2016). Charge transport and localization in atomically coherent quantum dot solids. Nat. Mater..

[6] Lan X (2020). Quantum dot solids showing state-resolved band-like transport. Nat. Mater..

[7] Abelson A (2020). Collective topo-epitaxy in the self-assembly of a 3D quantum dot superlattice. Nat. Mater..

[8] Lee J-S, Kovalenko MV, Huang J, Chung DS, Talapin DV (2011). Band-like transport, high electron mobility and high photoconductivity in all-inorganic nanocrystal arrays. Nat. Nanotechnol..

[9] Choi J-H (2012). Bandlike transport in strongly coupled and doped quantum dot solids: a route to high-performance thin-film electronics. Nano Lett..

[10] Wang ZM, Holmes K, Mazur YI, Salamo GJ (2004). Fabrication of (In, Ga)As quantum-dot chains on GaAs(100). Appl. Phys. Lett..

[11] Kunets VP (2013). Electron transport in quantum dot chains: dimensionality effects and hopping conductance. J. Appl. Phys..

[12] Hanson R, Kouwenhoven LP, Petta JR, Tarucha S, Vandersypen LMK (2007). Spins in few-electron quantum dots. Rev. Mod. Phys..

[13] Hsieh C-Y, Shim Y-P, Hawrylak P (2012). Theory of electronic properties and quantum spin blockade in a gated linear triple quantum dot with one electron spin each. Phys. Rev. B.

[14] Filikhin I, Matinyan SG, Vlahovic B (2012). Electron tunneling in double quantum dots and rings. J. Phys. Conf. Ser..

[15] Kuo DMT, Chang Y (2014). Long-distance coherent tunneling effect on the charge and heat currents in serially coupled triple quantum dots. Phys. Rev. B.

[16] Kagan MY, Valkov VV, Aksenov SV (2017). Effects of anisotropy and Coulomb interactions on quantum transport in a quadruple quantum-dot structure. Phys. Rev. B.

[17] Gong W, Zheng Y, Liu Y, Lü T (2006). Well-defined insulating band for electronic transport through a laterally coupled double-quantum-dot chain: nonequilibrium Green’s function calculations. Phys. Rev. B.

[18] Skibinsky-Gitlin ES, Rodríguez-Bolívar S, Califano M, Gómez-Campos FM (2019). Band-like electron transport in 2D quantum dot periodic lattices: the effect of realistic size distributions. Phys. Chem. Chem. Phys..

[19] Anderson PW, Thouless DJ, Abrahams E, Fisher DS (1980). New method for a scaling theory of localization. Phys. Rev. B.

[20] Ryndyk D, Gutierrez R, Song B, Cuniberti G, Burghardt I, May V, Micha D, Bittner E (2009). Green Function Techniques in the Treatment of Quantum Transport at the Molecular Scale. Green function techniques in the treatment of quantum transport at the molecular scale.

[21] Landauer R (1957). Spatial variation of currents and fields due to localized scatterers in metallic conduction. IBM J. Res. Dev..

[22] Whitney RS (2015). Finding the quantum thermoelectric with maximal efficiency and minimal entropy production at given power output. Phys. Rev. B.

[23] Liu Y, Zheng Y, Gong W, Gao W, Lü T (2007). Electronic transport through a quantum dot chain with strong dot-lead coupling. Phys. Lett. A.

[24] Brown PR (2014). Energy level modification in lead sulfide quantum dot thin films through ligand exchange. ACS Nano.

[25] Lundh F, Lundh F (2001). Python Standard Library.

[26] Williams, T., Kelley, C. *et al*. Gnuplot 5.2: an interactive plotting program. <http://www.gnuplot.info/> (2019).

[27] Abrahams E, Anderson PW, Licciardello DC, Ramakrishnan TV (1979). Scaling theory of localization: absence of quantum diffusion in two dimensions. Phys. Rev. Lett..

