# Supplementary material for: Quantum transport in a chain of quantum dots with inhomogeneous size distribution and manifestation of 1D Anderson localization
Source: Sci Rep. 2020 Oct 7;10:16701. doi: 10.1038/s41598-020-73578-z (PMC7541618; doi:10.1038/s41598-020-73578-z)
Supplement: Supplementary file 1 — Supplementary file1 [file 41598_2020_73578_MOESM1_ESM.docx]

**Supplementary Information**

Quantum transport in a chain of quantum dots with inhomogeneous size distribution and manifestation of 1D Anderson localization

Moon-Hyun Cha^1,2^ and Jeongwoon Hwang^3,^*

^1^School of Engineering, Brown University, Providence, RI 02912, USA

^2^CSE Team, Data & Information Technology Center, Samsung Electronics, Hwaseong 18448, Republic of Korea

^3^Department of Physics Education, Chonnam National University, Gwangju 61186, Republic of Korea

*Corresponding author: [phyjhwang@jnu.ac.kr](mailto:phyjhwang@jnu.ac.kr)


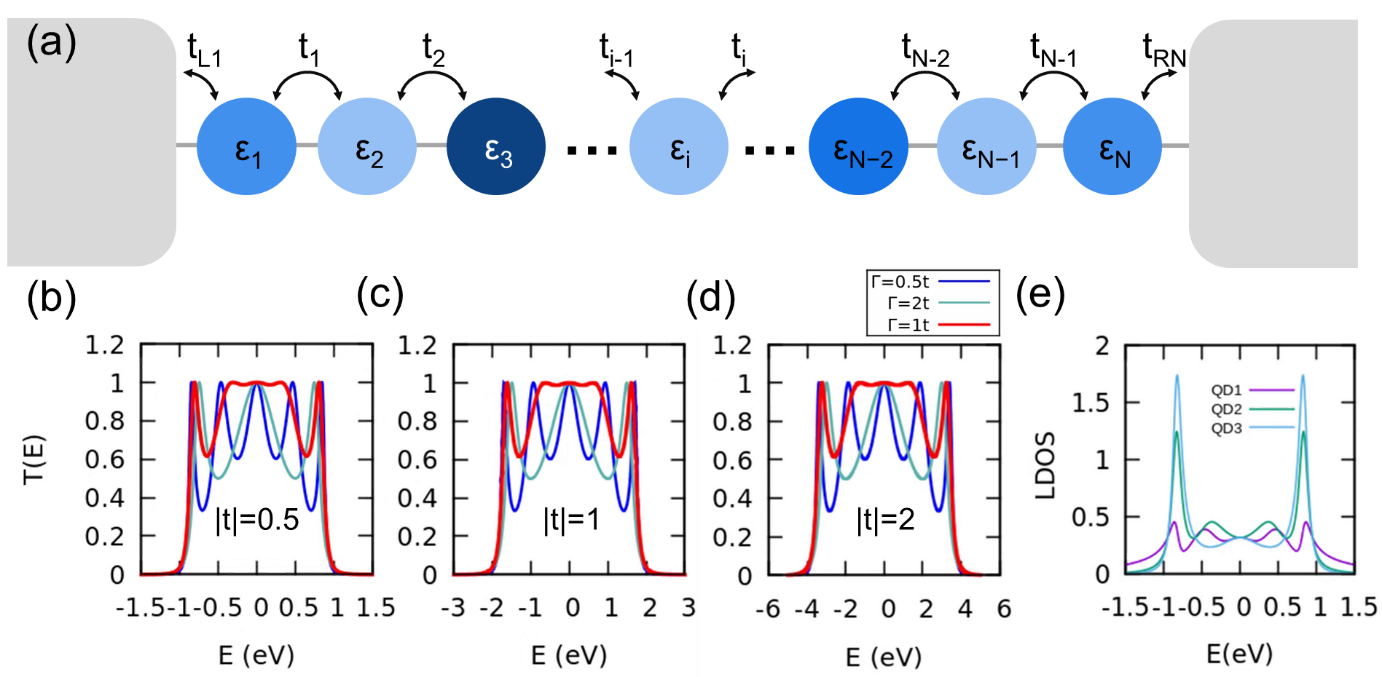


Fig. S1. (a) Schematic description of a 1D chain of N QDs in contact with two metallic leads. Different colors represent different energies (i.e., sizes). (b)-(d) Dependence of hopping parameter and dot-lead coupling on transmission probability of uniform 5-QDC. (e) Local density of states of 5-QD chain with |t|=0.5 projected on each dot (left half and the middle ones are chosen since the LDOS is symmetric). The magnitude of $t_{0}$ only affects the band width in a uniform manner, that is, the width of calculated transmission functions are uniformly scaled in energy as shown in the Figs. S1(b)-(d). We also calculate the local density of states (LDOS) of each QD for |t|=0.5, which is symmetric with respect to the center of the chain. LDOS of all QDs are well overlapped with each other in all energy range, which indicates that electronic states are well delocalized in the QDC.


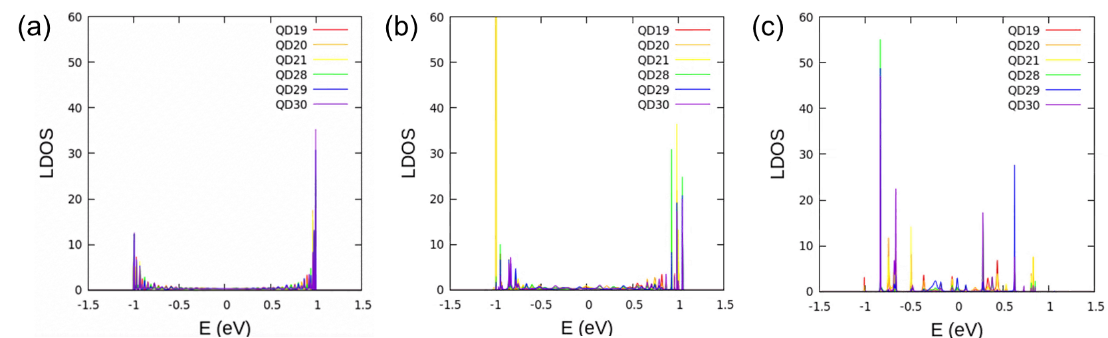


Fig. S2. LDOS of arbitrarily chosen 50-QDCs with (a) *σ*_ε_ = 0.02, (b) *σ*_ε_ = 0.1, and (c) *σ*_ε_ = 0.3_._ In (c), LDOS overlap only occurs among adjacent QDs, that is, QD19-21 and QD28-30. For large *σ*_ε_, electronic states are localized over a few QDs, which is presented as localized peaks in LDOS or overlap of LDOS (projected on each QD) only between adjacent QDs. This result is consistent with the prediction that asymmetry in energy levels of double QD results in localization of electronic states.^1^ While localized electronic states with similar energies can be overlapped in small systems, they can be far apart from each other without overlap of tails in large systems, as showing exponential decay of conductance with system size.^2^ For small *σ*_ε_, the LDOS spectra of QDs are similar to that of uniform QDCs (see Fig. S1(e) and Fig. S2(a)) as showing good overlap with each other originated from formation of extended states; this is the reason why QD solids can exhibit high-mobility band-like transport.


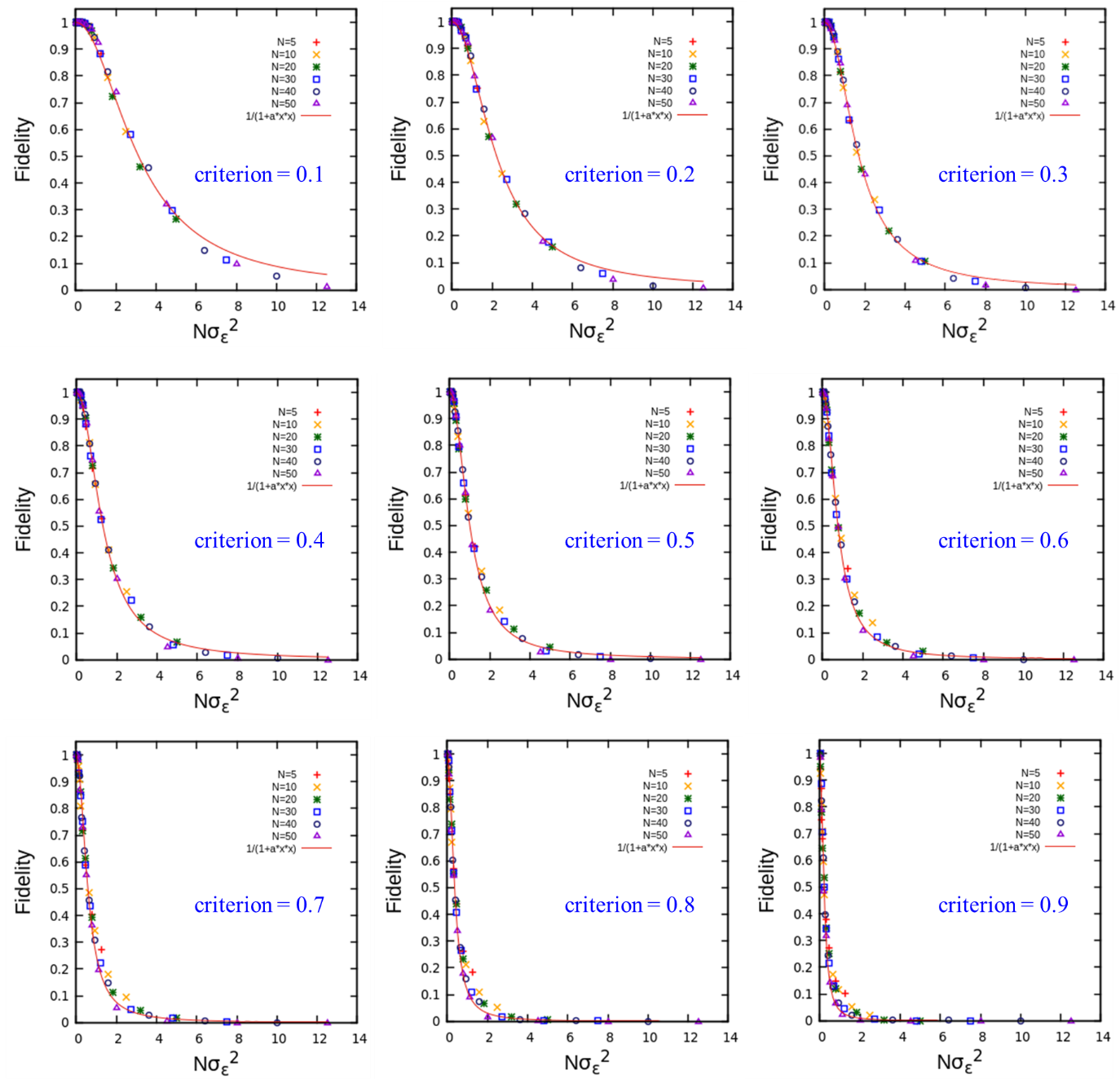


Fig. S3. Fidelity analysis with different choices of criterion value (from 0.1 to 0.9). As increasing the criterion, the fidelity function gets sharper but it retains the Lorentzian shape with a variable $N\sigma_{\epsilon}^{2}$. The calculated results are fitted to a function $f\left( N,\sigma_{\epsilon} \right)=\frac{1}{1+a\left( N{\sigma_{\epsilon}}^{2} \right)^{2}}$, where $a$ is a fitting parameter and $a$=1 when the criterion is 0.5. With a different choice of the criterion value, the fidelity can still be predicted by the same relation but scaled by the fitting parameter $a$.


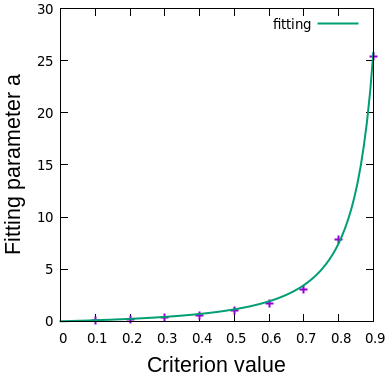


Fig. S4. The fitting parameters obtained in Fig. S3 are fitted to a function $a\left( c \right)=\frac{1}{2}\cdot\left( \frac{1}{\left( 1-c \right)^{b}}-1 \right),$and b=1.72 gives a generally good result. With a chosen criterion value c (0 < c < 1), the fidelity of any *N*-QDC can be predicted by $f\left( N,\sigma_{\epsilon} \right)=\frac{1}{1+{a(c)\left( N{\sigma_{\epsilon}}^{2} \right)}^{2}}$. When the criterion value goes to 1, the fitting parameter a(c) diverges. Physical intuition suggests that in a limiting case of $c\to0$, the fidelity should be 1. Also, in another limiting case of $c\to1$, the fidelity can be either 1 (if $\sigma_{\epsilon}=0$) or 0 (for any finite $\sigma_{\epsilon}$). Thus, the found function $a\left( c \right)$ is in a good agreement with the intuition.


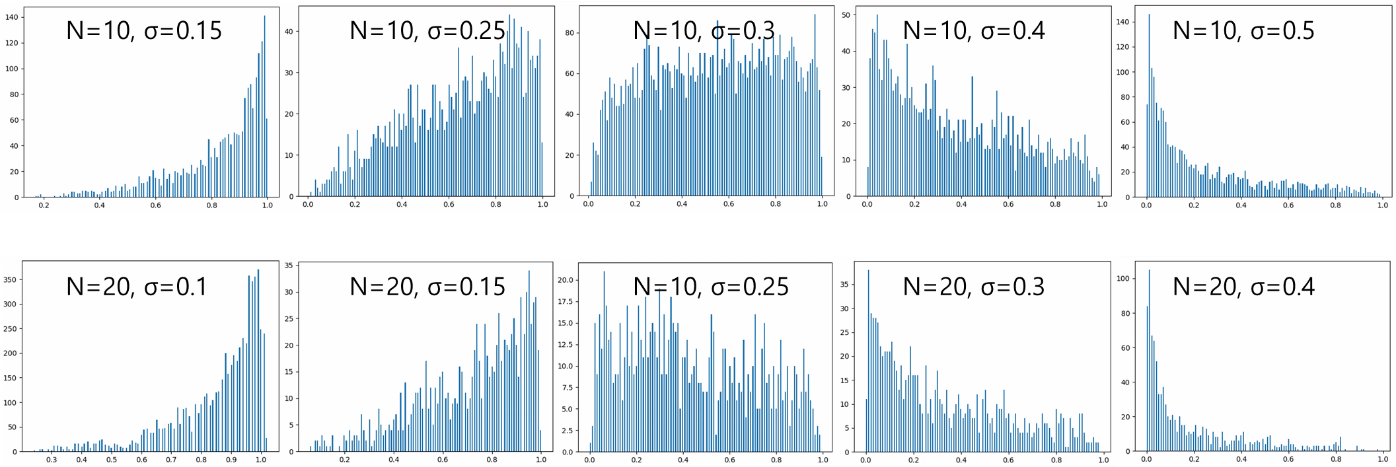


Fig. S5. Distribution of conductance measure $\bar{T}$ defined in the main text is represented as histogram for each pair of N and *σ*_ε_. From the above histogram, $\sigma_{\varepsilon}=\frac{1}{\sqrt{N}}$ is estimated to be the transition point where the majority of ensemble population is reversed based on the median value $\bar{T}=0.5$, and at this point population of ensemble members are approximately symmetric with respect to $\bar{T}=0.5$.


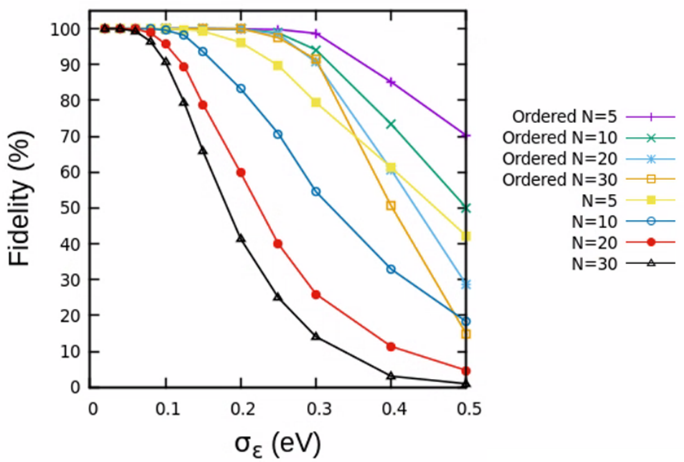


Fig. S6. Fidelity versus *σ*_ε_ graphs of ordered QDCs in increasing size are plotted with that of randomly ordered QDCs for *N* = 5-30. The fidelity decreases much slowly with the *σ*_ε_ compared to the randomly arranged case. This result indicates that the energy level difference between coupled QDs (i.e. coupled by tunneling matrix element) is important parameter for modulating transport capability.

**References**

1. Filikhin, I., Matinyan, S. G. & Vlahovic, B. Electron tunneling in double quantum dots and rings. *J. Phys. Conf. Ser.* **393**, 12012 (2012).

2. Abrahams, E., Anderson, P. W., Licciardello, D. C. & Ramakrishnan, T. V. Scaling Theory of Localization: Absence of Quantum Diffusion in Two Dimensions. *Phys. Rev. Lett.* **42**, 673–676 (1979).
